# The epidemiological features of respiratory tract infection using the multiplex panels detection during COVID-19 pandemic in Shandong province, China

**DOI:** 10.1038/s41598-023-33627-9

**Published:** 2023-04-18

**Authors:** Jie Zhang, Tao Yang, Mingjin Zou, Lili Wang, Lintao Sai

**Affiliations:** 1 Department of Clinical Laboratory, Qilu Hospital of Shandong University, 107 Wenhuaxi Road, Jinan, 250012 Shandong China; 2 Shandong Engineering Research Center of Biomarker and Artificial Intelligence Application, Jinan, Shandong China; 3 Department of Infectious Diseases, Qilu Hospital of Shandong University, Jinan, Shandong China

**Keywords:** Microbiology, Clinical microbiology, Infectious-disease diagnostics, Medical research, Epidemiology

## Abstract

Respiratory tract infection is one of the most common reasons for both morbidity and mortality worldwide. High attention has been paid to the etiological tracing of respiratory tract infection since the advent of COVID-19. In this study, we aimed to evaluate the epidemiological features of pathogens in respiratory tract infection, especially during COVID-19 pandemic. A total of 7668 patients with respiratory tract infection who admitted to Qilu Hospital of Shandong University from March 2019 to Dec 2021 were retrospectively included. The respiratory tract specimens were detected using a commercial multiplex PCR-based panel assay for common respiratory pathogens including influenza A virus (Flu-A), influenza A virus H1N1 (H1N1), influenza A virus H3N2 (H3N2), influenza B virus (Flu-B), parainfluenza virus (PIV), respiratory syncytial virus (RSV), adenovirus (ADV), Boca virus (Boca), human Rhinovirus (HRV), Metapneumovirus (MPV), Coronavirus (COV), Mycoplasma pneumoniae (MP), and Chlamydia (Ch). The positive rates were compared using a chi-square test. Compared with 2019, the positive rate of pathogen detection during from January 2020 to December 2021 was significantly lower, especially the detection of Flu-A. The positive rate of respiratory pathogen strains was 40.18% during COVID-19 pandemic, and a total of 297 cases (4.69%) of mixed infection with two or more pathogens were detected. There was no statistical difference in the positive rate between male and female patients. However, the positive rates of infection were different among different age groups, with higher incidence of RSV in infancy and toddler group, and MP infection in children and teenager group. While, HRV was the most common pathogen in the adult patients. Moreover, Flu-A and Flu-B were higher in winter, and MP and RSV were higher in spring, autumn and winter. The pathogens such as ADV, BOCA, PIV, and COV were detected without significant seasonal distribution. In conclusion, respiratory pathogen infection rates may vary by age and season, regardless of gender. During the COVID-19 epidemic, blocking transmission routes could help reduce the incidence of respiratory tract infection. The current prevalence of respiratory tract infection pathogens is of great significance for clinical prevention, diagnosis and treatment.

## Introduction

Respiratory tract infection (RTI) is one of the most common reasons for both mortality and morbidity worldwide, which can be categorized into upper and lower respiratory tract infection^1^. Although the etiology of different RTI is diverse, some serious complications such as pneumonia and myocarditis may happen on the patients, especially among the children and elderly, resulting in heavy burden on individuals and society. Moreover, the lifestyle has been dramatically changed since the advent of COVID-19, especially with the prevention of wearing masks, washing hands frequently. The pathogens affecting respiratory tract infection might be also changed. To investigate the pathogen epidemiology of RTI is critical for clinic diagnosis, treatment, and prognosis.

The upper respiratory tract infection (URTI) usually occurs with short, mild and self-limited process^2^. The high incidence of URTI is usually caused by viruses which can target the human respiratory tract, and cause different clinical manifestations, even lead to serious complications like acute respiratory distress syndrome (ARDS)^3–6^. The lower respiratory tract infection (LRTI) is mainly induced by virus, mycoplasma, chlamydia and bacteria, etc. In clinic, different pathogens may cause similar symptoms^7–9^. It’s difficult to identify patient diseases because of the overlap or similar clinical presentations. For example, mycoplasma pneumoniae (MP) and respiratory syncytial virus (RSV) can both cause symptoms such as cough, runny nose, nasal congestion and fever in patients. In recent years, due to the extensive application of antibiotics, the trend of respiratory tract infection is increasing year by year. Strong infectivity and short incubation period are the obvious clinical characteristics of respiratory tract infection, especially for children and the elderly, which may lead to pneumonia, myocarditis and other complications. In addition, the epidemic forms and outbreak seasons of respiratory pathogens can be shown with a great diversity across different regions, population demography, and seasons^10^. Evermore, during the COVID-19 pandemic, people's lifestyle has changed greatly, and the pathogens affecting respiratory tract infection have also been changed. Therefore, understanding the infection of respiratory pathogens in different seasons and different age groups in this region has a good guiding role for early clinical diagnosis, treatment and medication.

Over the last decades, the diagnostic work up of clinical infections has been changed tremendously, especially with the rapid development of molecular diagnostic methods^11,12^. The multiplex polymerase chain reaction (PCR) assay has been confirmed as a better tool in the clinical detection of potential pathogens. In this study, we retrospectively reviewed the patients with RTI in Qilu Hospital of Shandong University from 2019 to 2021 who were identified pathogens using a multiplex PCR assay platform. We also evaluated the epidemiological features of respiratory pathogens infection during COVID-19 pandemic including aging, seasonal variations of different pathogens.

## Results

### Demographic data

In 2019, a total of 1334 patients were included, and 823 respiratory pathogen strains were detected, with a total positive rate of 61.69%. In addition, from January 2020 to December 2021, a total of 6334 patients including 2512 females and 3822 males with RTI screening were recruited in this study, and the patients were grouped by age (Table 1). A total of 2545 respiratory pathogen strains were detected, with a positive rate of 40.18% during the COVID-19 pandemic. Compared with the year of 2019, the overall positive rate of respiratory pathogens after January 2020 was significantly lower (*P* < 0.01).

**Table 1:** Demographic, clinical data of patients enrolled before and after COVID-19.

Variable	% (No. of patients in 2019)	% (No. of patients in 2020 and 2021)
Gender		
Female	59.54 (365/613)	37.98 (954/2512)
Male	59.36 (428/721)	41.63 (1591/3822)
Age group		
0–6 months	72.61 (114/157)	52.02 (245/471)
7–24 months	87.68 (185/211)	57.28 (618/1079)
3–6 years	75.09 (199/265)	48.67 (715/1469)
7–18 years	37.92 (113/298)	40.35 (443/1098)
19–35 years	31.63 (31/98)	21.55 (92/427)
36–60 years	44.09 (56/127)	19.57 (156/797)
> 60 years	53.37 (95/178)	27.79 (276/993)
Detection of pathogens		
Single pathogen infection	61.69 (823/1334)	40.18 (2545/6334)
Mixed pathogens infection	7.05 (94/1334)	4.69 (297/6334)

### Comparison of individual pathogen infection

**Figure 1:**
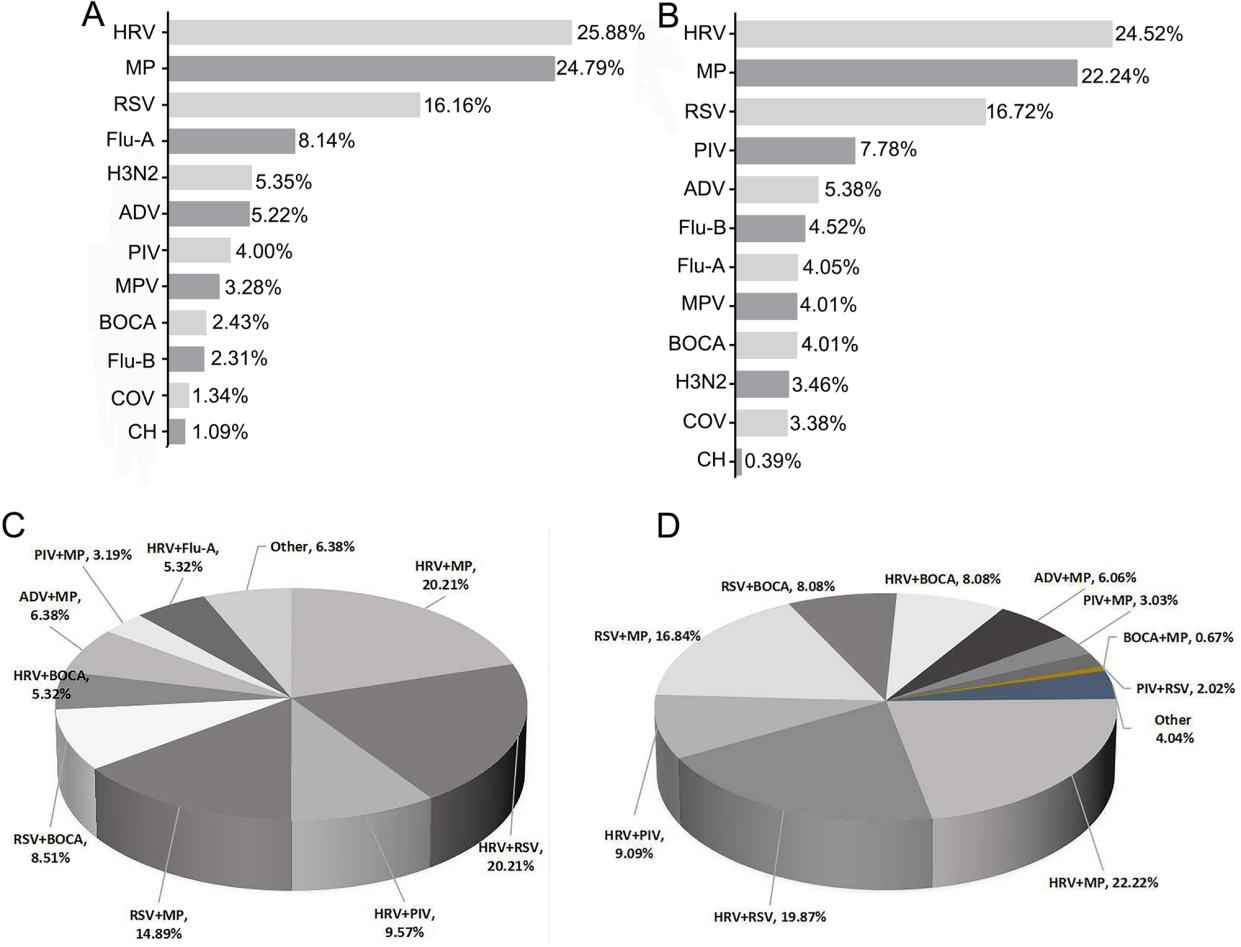
Detection of respiratory pathogens infection before and after the COVID-19 pandemic. (**A**) The overall positive rates of pathogens in 2019. (**B**) The overall positive rates of pathogens in 2020 and 2021. (**C**) The co-infection rate among patients in 2019. (**D**) The co-infection rate of pathogens in 2020 and 2021. *HRV* human Rhinovirus, *MP* mycoplasma pneumoniae, *RSV* respiratory syncytial virus, *Flu-A* influenza A virus, *Flu-B* influenza B virus, *H3N2* influenza A virus H3N2, *PIV* parainfluenza virus, *ADV* adenovirus, *Boca* Boca virus, *MPV* Metapneumovirus, *HCOV* Coronavirus, *Ch* Chlamydia.

Among patients in 2019, the most commonly detected pathogen was HRV (25.88%, 213/823), followed by MP (24.79%, 204/823), RSV (16.16%, 133/823), Flu-A (8.14%, 67/823), H3N2 (5.35%, 44/823), ADV (5.22%, 43/823), PIV (4.00%, 33/823), MPV (3.28%, 27/823), BOCA (2.43%, 20/823), Flu-B (2.31%, 19/823), COV (1.34%, 11/823), CH (1.09%, 9/823). During the COVID-19 pandemic, the overall positive rate was lower, and the top three common pathogens were still HRV (24.52%, 624/2545), MP (22.24%, 566/2545), and RSV (16.27%, 414/2545). To be noted, the detection rates of Flu-A (4.05%, 103/2545) and H3N2 (3.46%, 88/2545) decreased significantly compared with 2019 (*P* < 0.01). In 2020–2021, Flu-A and Flu-B broke out simultaneously in early 2020, and then came with the prevalence rate of Flu-B. We found the increased positive rates of Flu-B (4.52%, 115/2545) and COV (3.38%, 86/2545) compared with 2019 (*P* < 0.01). There were also some statistical differences in the positive rates of CH (0.39%, 10/2545) infection (*P* < 0.05). The positive rates of other pathogens were ADV (5.38%, 137/2545), MPV (4.01%, 102/2545), BOCA (4.01%, 102/2545), and showed as similar as those in 2019 (Fig. 1A,B).

### Comparison of mixed-infection pathogens

The patients infected by two or more viruses (mixed infection) were further evaluated. The mixed-infection rate among patients in 2019 was 7.05% (94/1334). However, there were 297 cases of mixed-infection pathogens detected during COVID-19 pandemic, with a positive rate of 4.69% (297/6334). Among all the respiratory infection patients in 2019, 6 cases were infected with more than two pathogens (6.38%, 6/94) and 88 cases were infected with two pathogens (93.62%, 88/94). Among the mixed infection with two pathogens, HRV + MP 20.21%, 19/94) and HRV + RSV (20.21%, 19/94) were the most common pathogens in 2019, followed by HRV + PIV (9.57%, 9/94), RSV + MP (14.89%, 14/94), RSV + BOCA (8.51%, 8/94), ADV + MP (6.38%, 6/94), HRV + BOCA (5.32%, 5/94), HRV + Flu-A (5.32%, 5/94) and PIV + MP (3.19%, 3/94). During the COVID-19 pandemic, similar positive rates of infection were noted in samples, 12(4.04%,12/297) with more than two pathogens and 285 (95.96%,285/297) with only two pathogens. The most common pathogen combinations were HRV + MP (22.22%, 66/297) and HRV + RSV (19.87%, 59/297), followed by RSV + MP (16.84%, 50/297), HRV + PIV (9.09%, 27/297), RSV + BOCA (8.08%, 24/297), HRV + BOCA (8.08%, 24/297), ADV + MP (6.06%, 18/297), PIV + MP (3.03%, 9/297), PIV + RSV (2.02%, 6/297), BOCA + MP (0.67%, 2/297). Compared with 2019, the co-infection rates of HRV + Flu-A decreased significantly (*P* < 0.01), and the co-infection rates of HRV + MP and HRV + RSV showed no significant differences (Fig. 1C,D).

### Comparison of pathogen infection by gender

**Figure 2:**
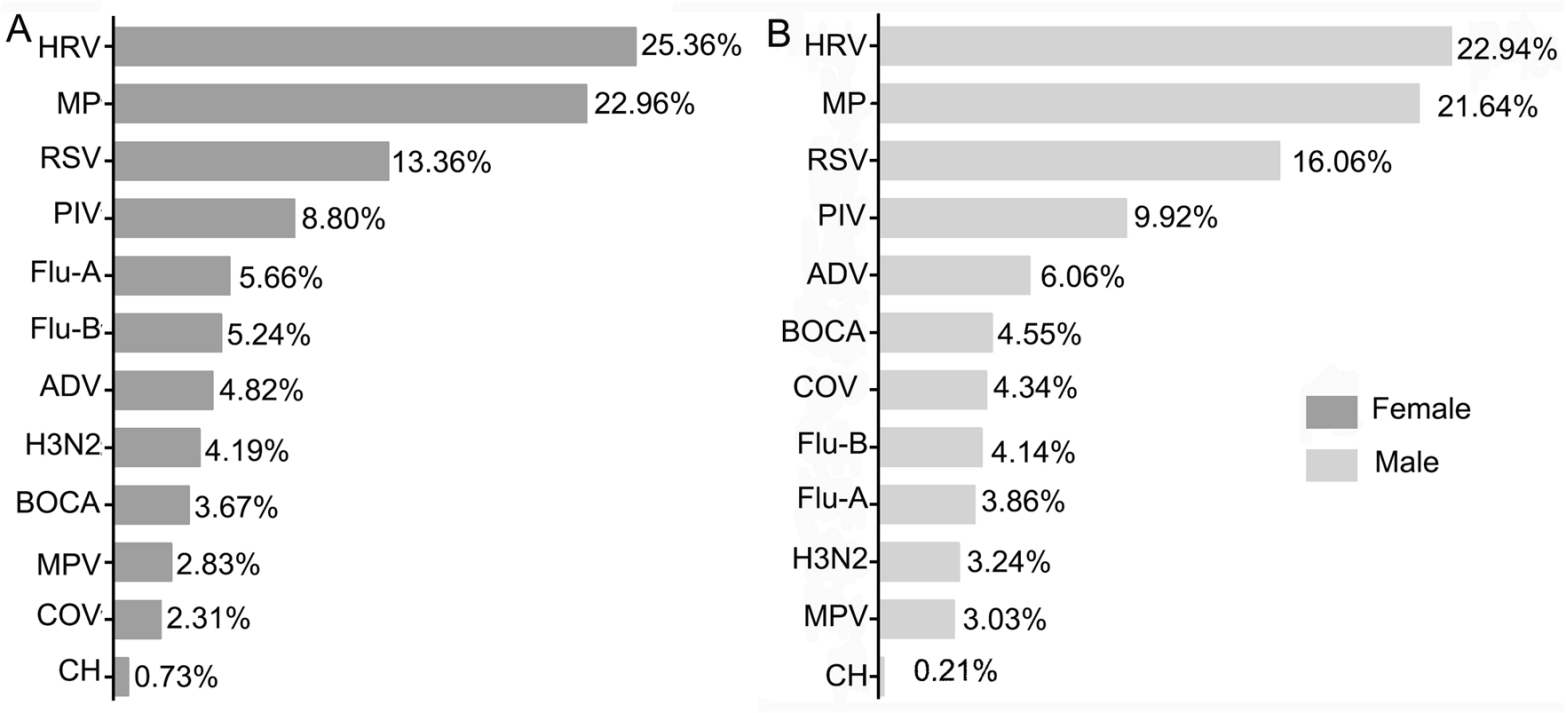
Detection of respiratory pathogens in men and women after the COVID-19 pandemic. (**A**) The overall positive rates of pathogens in male. (**B**) The overall positive rates of pathogens in female.

During the COVID-19 pandemic, the positive rate of pathogen infection was 38.77% (974/2512) in female and 41.10% (1571/3822) in male (Fig. 2). There was no statistical difference in the positive rate between male and female patients (χ^2^ = 3.42, *P* > 0.05). The highest infection rate of respiratory pathogens in the two groups of patients of different genders was HRV, followed by MP and RSV. There were also no significant differences in the positive rates of other respiratory pathogens between males and females.

### Comparison of pathogen infection by age

**Figure 3:**
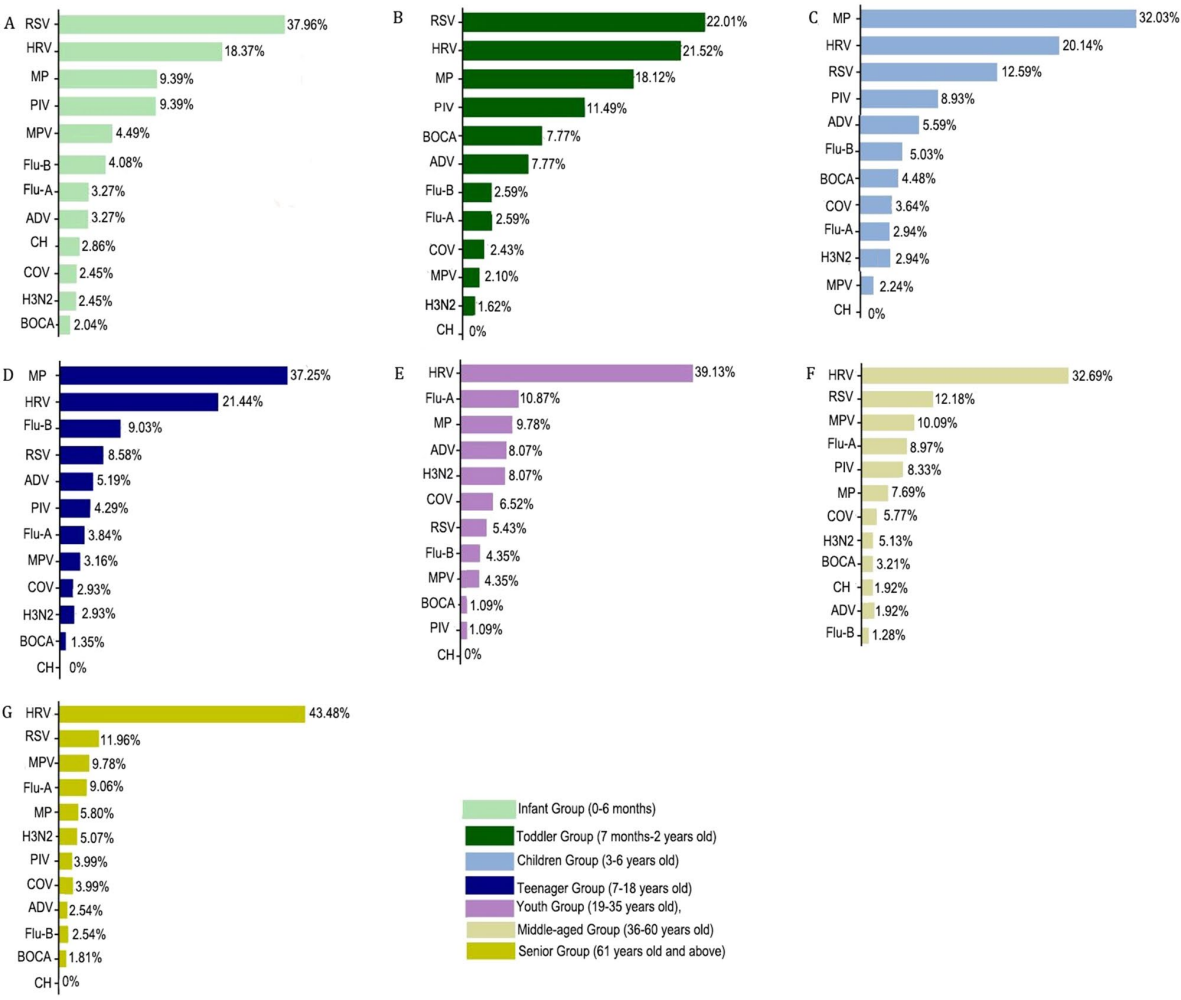
Detection of respiratory pathogens in different age groups after the COVID-19 pandemic. (**A**) The infant group. (**B**) The toddler group. (**C**) The children group. (**D**) The teenager group. (**E**) The youth group. (**F**) The middle-aged group. (**G**) The senior group.

In current study, all patients were divided into 7 groups according to different age. The results showed that the positive rates of infant group (0–6 months) and toddler group (7 months-2 years old) during the COVID-19 pandemic were significantly higher than those of other groups (*P* < 0.01). As shown in Fig. 3, among all the detected pathogens, the rates of RSV were higher in infant group and toddler group (*P* < 0.05), which were 37.96% (93/245) and 22.01% (136/618) respectively, followed by HRV (18.37%, 45/245 and 21.52%, 133/618), MP (9.39%, 23/245 and 18.12%, 112/618) and PIV (9.39%, 23/245 and 11.49%, 71/618). In the children group, the higher positive rates of respiratory pathogens were MP and HRV (32.03%, 229/715 and 20.14%, 144/715, respectively), however, the positive rate of RSV became lower (12.59%, 90/715). In the teenager group, the highest incidence pathogen was MP (37.25%, 165/443), followed by HRV (21.44%, 95/443), Flu-B (9.03%, 40/443), and RSV (8.58%, 38/443). In addition, HRV was found with higher incidence in the youth, middle-aged and senior groups.

### Comparison of seasonality

**Figure 4:**
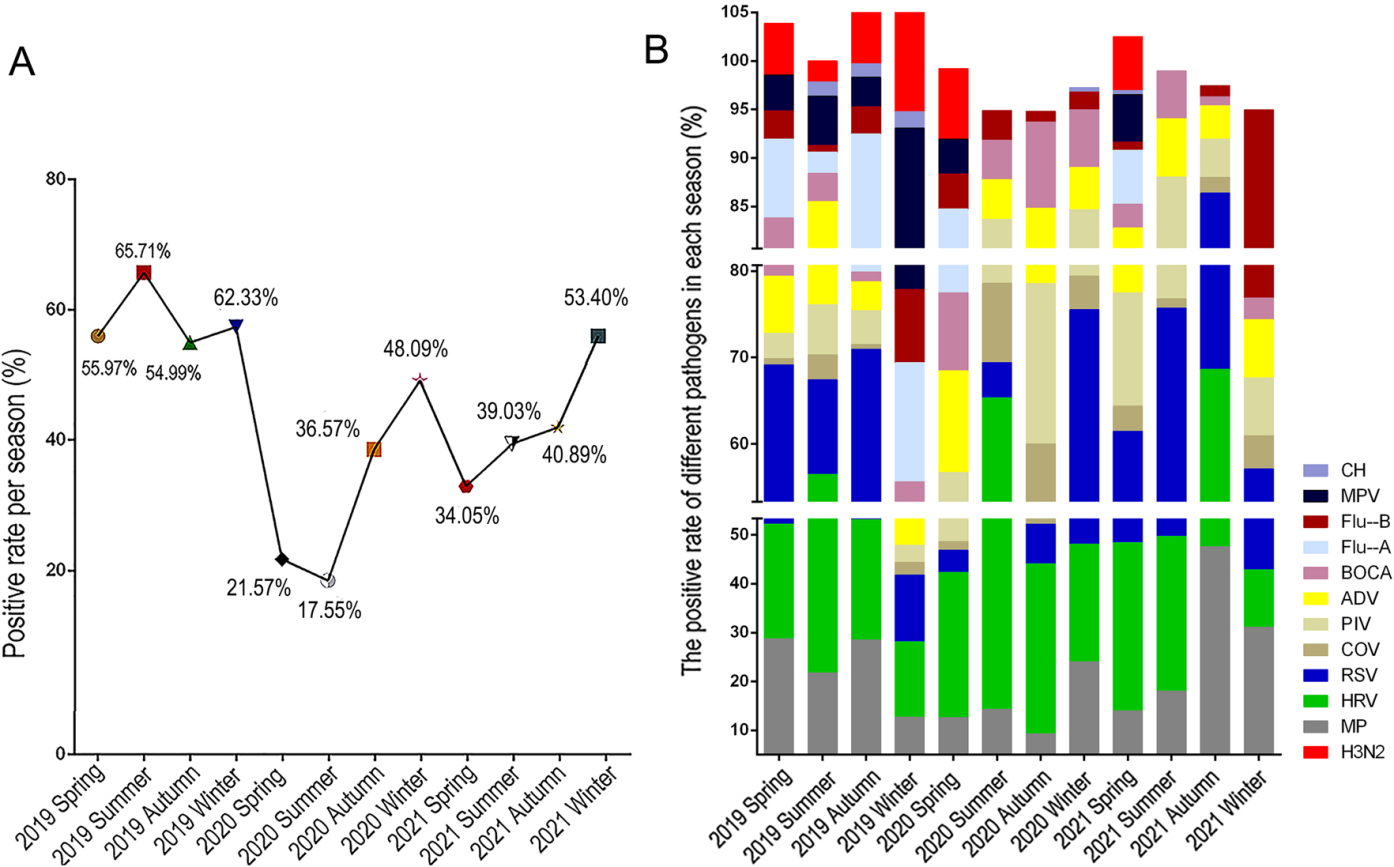
Detection of respiratory pathogens in different seasons after the COVID-19 pandemic. (**A**) The positive rates of pathogens in different seasons. (**B**) Seasonal distribution of positive detected pathogens during 2019 and 2021.

In 2019, the positive rates of respiratory pathogens were 55.97% (136/243) in spring of 2019, 65.71% (138/210) in summer of 2019, 54.99% (358/651) in autumn of 2019 and 62.33% (642/1030) in winter of 2019, respectively. However, after the COVID-19 occurrence, the total positive rate of respiratory pathogens was significantly lower. The positive rates of respiratory pathogens were 21.57% (110/510) in spring of 2020, 17.55% (93/530) in summer of 2020, 36.57% (256/700) in autumn of 2020 and 48.09% (4287/890) in winter of 2020, 34.05% (252/740) in spring of 2021, 39.03% (281/720) in summer of 2021, 40.89% (422/1032) in autumn of 2021 and 53.40% (220/412) in winter of 2021, respectively (Fig. 4A).

The seasonality variation of major pathogens could also be observed. For example, the positive rate of MP was the highest in spring of 2019, followed by HRV, RSV and Flu-A. In summer of 2019, the higher positive rate of pathogen was HRV, followed by MP, RSV, and ADV. In autumn of 2019, the higher positive rate of pathogen was MP, followed by HRV, RSV and Flu-A. In winter of 2019, the higher positive rate of pathogen was HRV, followed by MPV, Flu-A, and RSV. In 2020 and 2021, similar positive rates of HRV were noted in samples in spring and summer, followed by MP or RSV. In 2020, RSV had a higher positive rate in winter, followed by HRV, MP, and BOCA. In 2021, the positive rate of MP was the highest in winter, followed by Flu-B, RSV, and HRV (Fig. 4B). Among all the positive detected pathogens, HRV had a higher rate with no significant seasonal variation observed. Several pathogens such as ADV, BOCA, PIV, and COV did not differ significantly throughout seasons in our study.

## Discussion

Respiratory tract infection is considered as one of the most important health problems with an enormous impact on patients and society, which shows higher incidence especially in children and some immunocompromised adults with symptoms ranging from the common cold to pneumonia^13,14^. With the development of molecular diagnosis, the multiplex PCR assay provides a better tool as the routine diagnostic test. The current study assessed the distribution and epidemic characteristics of respiratory pathogens in Jinan after the outbreak of COVID-19 using a multiplex PCR panels assay.

The common infectious respiratory pathogens, such as Flu, RSV, PIV, hMPV, HRV, MP, H3N2, COV, ADV and BOCA, may be different with geographical variation or seasonality, and some pathogens may occur throughout the year with individual variation. Some pathogens can also cause severe disease in immunocompromised hosts and are highly susceptible to cross-infection. However, the pathogen spectrum might be changed with years, especially since the advent of COVID-19 with washing hands frequently and wearing masks. In the current study, we found that the positive rates of some common respiratory pathogens, such as Flu-A, H3N2, MP, and MPV, decreased significantly after 2019. However, the positive rate of HRV did not change significantly, which was similar with the detection of respiratory pathogens in Japan^15^.

In 2019, the positive rate of respiratory pathogen detection in Jinan was 61.69%. HRV, MP, RSV and Flu-A were frequently identified. From January 2020 to December 2021, the positive rate of respiratory pathogens was 40.18%, which was significantly lower than that in 2019, and the positive rates of PIV and Flu-A were found to be significantly lower in single pathogen detection. We found that the prevalence of Flu-A decreased and that of Flu-B increased slightly in 2020–2021, with similar trend of influenza virus transmission around the world during the COVID-19 period. The dominant transmission for respiratory pathogens is the direct or indirect exposure to droplets or contact with contaminated surfaces produced by infected individuals^16^. After the COVID-19 pandemic, the public was encouraged with the wearing of masks, regular hand washing and frequent disinfection, which greatly reduced the transmission of respiratory pathogens. In this study, we found that the highest positive rate of pathogen detection was HRV, which was consistent with the results of other studies^17^. However, there was no significant different of RSV detection rate compared with 2019. HRV is a non-enveloped virus and not easily inactivated by ethanol-containing disinfectants. Therefore, despite the widespread use of ethanol-based hand sanitizers during the COVID-19 pandemic, the viral properties of HRV make it impossible to remove rhinoviruses from the skin using conventional hand sanitizers^18^.

During the COVID-19 pandemic, the co-infection rate of respiratory pathogens was significantly decreased, but the common models were still HRV and MP co-infection, or HRV and RSV co-infection. We found that HRV was not only most frequently detected in single pathogen infection, but also in combination with other pathogens, which was accounted for more than half of co-infections during 2019 and the COVID-19 pandemic. The positive rates of HRV combination with MP, RSV, or BOCA were not decreased, while the positive rate of HRV combined with Flu-A was decreased significantly. Anner et al. had found a significant positive correlation between HRV and PIV, HRV and RSV, and HRV and ADV. There was a negative correlation between HRV and influenza A virus. These may be due to geographic location, seasonality, or host responses, which requires further study in the future^19^. The other possible reason of HRV prevalence may be associated with the weakened competitive inhibitory effect of other common respiratory viruses on rhinovirus, and less affection of transmissibility with common prevention methods. In general, HRV is the most common cause of upper respiratory tract disease, and has now been found to be associated with worsening chronic disease, asthma development, severe bronchiolitis in infants and children, and also fatal pneumonia in the elderly and some immunocompromised adults. A better understanding of the mechanisms leading to HRV infection and the role of the host immune response may be helpful for early prevention, diagnosis and treatment^20^.

In this study, 2512 female patients and 3822 male patients were tested during the COVID-19 pandemic, and there was no significant difference in the positive rate and pathogen detection. However, some significant variation was noted in different age groups. We found that the positive rates of pathogens in the infant and toddler groups were significantly higher than those of other groups. The top three pathogens detected were RSV, HRV and MP, which were consistent with the previous study^21^. Moreover, the positive rates of pathogens in children and teenagers were lower than those in infants and toddler children, but much higher than those in youth, middle-aged and elderly groups. The highest pathogen detection rate was MP in children and teenager groups. MP is one of the most common pathogens causing community-acquired respiratory infections, which can be transmitted and spread especially in the group aged 3–18 because of the closed or semi-closed environments in schools^22^. Our data showed that the positive detection rate of MP was 24.79% in 2019, and decreased after the COVID-19 pandemic, which suggested that effective public interventions and restricting close contact could reduce the spread of MP. In addition, HRV, MP, and RSV were more common in the youth, middle-aged and elderly groups. Similar to other studies, the positive rate of respiratory pathogens was significantly lower in the young, middle-aged and elderly groups, especially in the youth and middle-aged groups^23^.

The current study also assessed the possible variation with the seasonal variability. Pandemic influenza is a major public health problem worldwide. Influenza mostly occurs in winter and disappears slowly in spring and summer. RSV is also a seasonal virus that occurs annually during the winter in temperate climates and the rainy season in tropical climates. The positive rate of RSV in winter in 2019 and 2020 was significantly higher than that in other seasons. In 2019, the positive rate of RSV was around 16.16%. However, HRV had a higher positive rate with no significant seasonal variation. During the COVID-19 pandemic, the RSV detection rate decreased to 4.5% in the spring of 2020. This is not only related to the seasonal outbreak of RSV, but also may related to some hygiene habits.

## Conclusion

The current study showed that from January 2020 to December 2021, the positive rate of respiratory pathogens was lower than that in 2019. The most common viruses in Jinan were HRV, RSV and MP with high incidence in recent years. During the epidemic of respiratory viruses, analyzing the prevalence of respiratory tract infection pathogens in Jinan is of great significance for clinical diagnosis and treatment. It can help clinicians to take targeted preventive measures, reduce the abuse of antibiotics, shorten the time of medical treatment and improve the efficiency of medical treatment.

## Material and methods

### Study design and population

The current retrospective study was conducted in Qilu hospital of Shandong University, a regional medical centers with patients from different cities in Shandong Province. In 2019, a total of 1334 patients with RTI screening were enrolled. In addition, from January 2020 to December 2021, a total of 6334 patients were further enrolled and divided to 7 age groups: infant group (0–6 months), toddler group (7 months-2 years old), children group (3–6 years old), teenager group (7–18 years old), youth group (19–35 years old), middle-aged group (36–60 years old), senior group (61 years old and above). Specimens of nasopharyngeal swab (NPS), throat swab (TS) or bronchoalveolar lavage (BAL) were collected for further detection. The study was approved by the ethics committee of Qilu Hospital of Shandong University and all methods were performed in accordance with the Declaration of Helsinki and the relevant guidelines and regulations.

### The detection of respiratory tract pathogens

The nucleic acids of specimens were extracted by the automated nucleic acid extraction system (Nucleic Acid Extraction or Purification Kit, HEALTH GENETECH, Ningbo, China). A total of 13 respiratory tract pathogens, including influenza A virus (Flu-A), influenza A virus H1N1 (H1N1), influenza A virus H3N2 (H3N2), influenza B virus (Flu-B), parainfluenza virus (PIV), adenovirus (ADV), Boca virus (Boca), human Rhinovirus (HRV), Metapneumovirus (MPV), Coronavirus (COV), Chlamydia (Ch), MP and RSV, were performed with relevant guideline by detecting the fluorescent signals of different fragments using a multiplex panel assay (Multiplex detection of 13 respiratory tract pathogens with PCR capillary electrophoresis, HEALTH GENETECH, Ningbo, China).

### Data analysis

All statistical analysis were performed using SPSS 19.0 software. Pearson’s χ^2^ or Fisher’s exact test were used to compare the category variables between groups when appropriate, and *P* < 0.05 was considered statistically significant.

### Ethical approval

The study was approved by the ethics committee of Qilu Hospital of Shandong University. Written informed consent was acquired from every enrolled patients or their guardians.

## Data Availability

All data generated or analyzed during this study are included in this published article.
